# Case report and literature review: thyroid carcinoma showing intrathyroid thymic carcinoma

**DOI:** 10.3389/fonc.2022.923683

**Published:** 2022-08-05

**Authors:** Ye Yuan, Changshu Ke, Guopeng Zhang, Jun Zhang, Qianxia Li

**Affiliations:** ^1^ Department of Gastroenterology, Tongji Hospital, Tongji Medical College, Huazhong University of Science and Technology, Wuhan, China; ^2^ Department of Pathology, Tongji Hospital, Tongji Medical College, Huazhong University of Science and Technology, Wuhan, China; ^3^ Department of Nuclear Medicine, Tongji Hospital, Tongji Medical College, Huazhong University of Science and Technology, Wuhan, China; ^4^ Department of Geriatrics, Tongji Hospital, Tongji Medical College, Huazhong University of Science and Technology, Wuhan, China; ^5^ Department of Oncology, Tongji Hospital, Tongji Medical College, Huazhong University of Science and Technology, Wuhan, China

**Keywords:** case report, lenvatinib, literature review, intrathyroid thymic carcinoma (ITTC), tyrosine kinase inhibitor

## Abstract

**Background:**

Intrathyroid thymic carcinoma (ITTC) is a rare malignancy of the thyroid gland with histological and immunophenotypic resemblance to thymic carcinoma. Surgery combined with adjuvant radiotherapy improves the survival of patients with ITTC. However, for patients with extensive metastases, there is currently no effective treatment. Chemotherapy is an option but has not demonstrated improved patient survival.

**Methods and results:**

A female patient presented with metastases to the pleura, lung, and bone 16 years after surgery for ITTC. As radiotherapy and chemotherapy failed to control the recurrent disease, lenvatinib treatment was initiated. After 3 months, positron emission tomography/computed tomography showed a substantial reduction of all metastatic lesions and decreased tumor metabolism. The patient continues to receive lenvatinib and remains well and symptom-free.

**Conclusion:**

For patients with ITTC who have progressive, life-threatening metastases, lenvatinib represents a valuable salvage therapy that may offer a sustained reduction in tumor burden and maintenance of quality of life.

## Introduction

Intrathyroid thymic carcinoma (ITTC), previously known as carcinoma showing thymus-like differentiation (CASTLE), is a rare intrathyroidal neoplasm, probably arising from ectopic thymus or branchial pouch remnants (1). Intrathyroidal epithelial thymoma was first described by Miyauchi et al. (2). Subsequently, these tumors were classified by Chan and Rosai (3) into four groups according to their clinical and pathological features: ectopic hamartomatous thymoma, ectopic cervical thymoma, spindle epithelial tumor with thymus-like differentiation (SETTLE), and CASTLE. In the most recent edition of the World Health Organization classification of tumors of endocrine organs, ITTC was designated as an independent clinicopathologic entity of thyroid tumors (4, 5).

Due to the rarity of this disease, there is no clear definitive treatment strategy. Surgical resection of ITTC is usually attempted initially. Curative surgery followed by adjuvant radiotherapy should be considered in cases with extrathyroidal extension and nodal metastases. Although uncommon, there are reports of ITTC metastasizing to the brain, liver, and lungs (3). For patients with advanced disease, chemotherapy is an option but has not been shown to improve patient survival (6, 7).

Tyrosine kinase inhibitors (TKIs) that inhibit vascular endothelial growth factor receptor (VEGFR) signaling and tumor angiogenesis have been used to treat advanced, progressive, and radioactive iodine (RAI)-refractory thyroid cancers for the past decade. Lenvatinib is an oral multitargeted tyrosine kinase inhibitor of VEGFR, fibroblast growth factor receptor (FGFR), platelet-derived growth factor receptor (PDGFR)-α, KIT, and rearranged during transfection (RET). It has shown antitumor activity and received regulatory approval by the US Food and Drug Administration and European Medicines Agency for the treatment of patients with locally advanced or metastatic, progressive, differentiated thyroid cancer (DTC) (8–10). However, there are no available data on the use of lenvatinib for the treatment of ITTC.

Here, we report a case of ITTC with a favorable response to second-line systemic treatment with lenvatinib. The Institutional Review Board of Tongji Hospital approved this case study and the patient provided consent.

## Case presentation

A 38-year-old woman presented to our clinic in 2002 with a tumor in the inferior pole of the left thyroid that was considered malignant after a needle biopsy. The mass was located at the inferior pole of the thyroid and was approximately 3.0 × 2.5 cm in size. The capsule was intact and there was no extracapsular invasion or cervical lymph node metastasis. Thyroidectomy and functional neck dissection were performed 1 month later at Tongji Hospital. Postoperative pathology showed poorly differentiated squamous cell carcinoma. Due to limitations in techniques available at the time, immunohistochemical detection was not performed. The patient subsequently attended Vancouver General Hospital in Canada, where she was diagnosed with ITTC based on positive staining for pankeratin, CK5, CD5, CD117, and CD10. Radiotherapy was administered (60 Gy in 30 fractions) and the patient underwent follow-up computed tomography (CT) and positron emission tomography (PET) scans at BC Cancer Center and Tongji Hospital from 2003 to 2017 with no abnormal findings.

A routine follow-up examination at Vancouver General Hospital in 2017 showed thickening of the right pleura, and the needle biopsy pathology report suggested that the disease had recurred. The patient received local radiotherapy (50 Gy in 25 fractions). In 2018, progression of the local pleural lesions was observed, and radiotherapy was repeated at the same dose. A PET scan in January 2019 showed a poor response to radiotherapy. The right pleura continued to thicken, nodules were present in the right lung and right interlobular fissure, the second anterior rib cortex on the right was irregular, and the metabolism of all lesions was high.

In April 2019, the patient developed cough and dyspnea. An examination revealed right pleural effusion. She received chemotherapy (paclitaxel + carboplatin) for five cycles at Vancouver General Hospital. By the end of the last cycle in November 2019, the pleural fluid was controlled.

**Figure 1:**
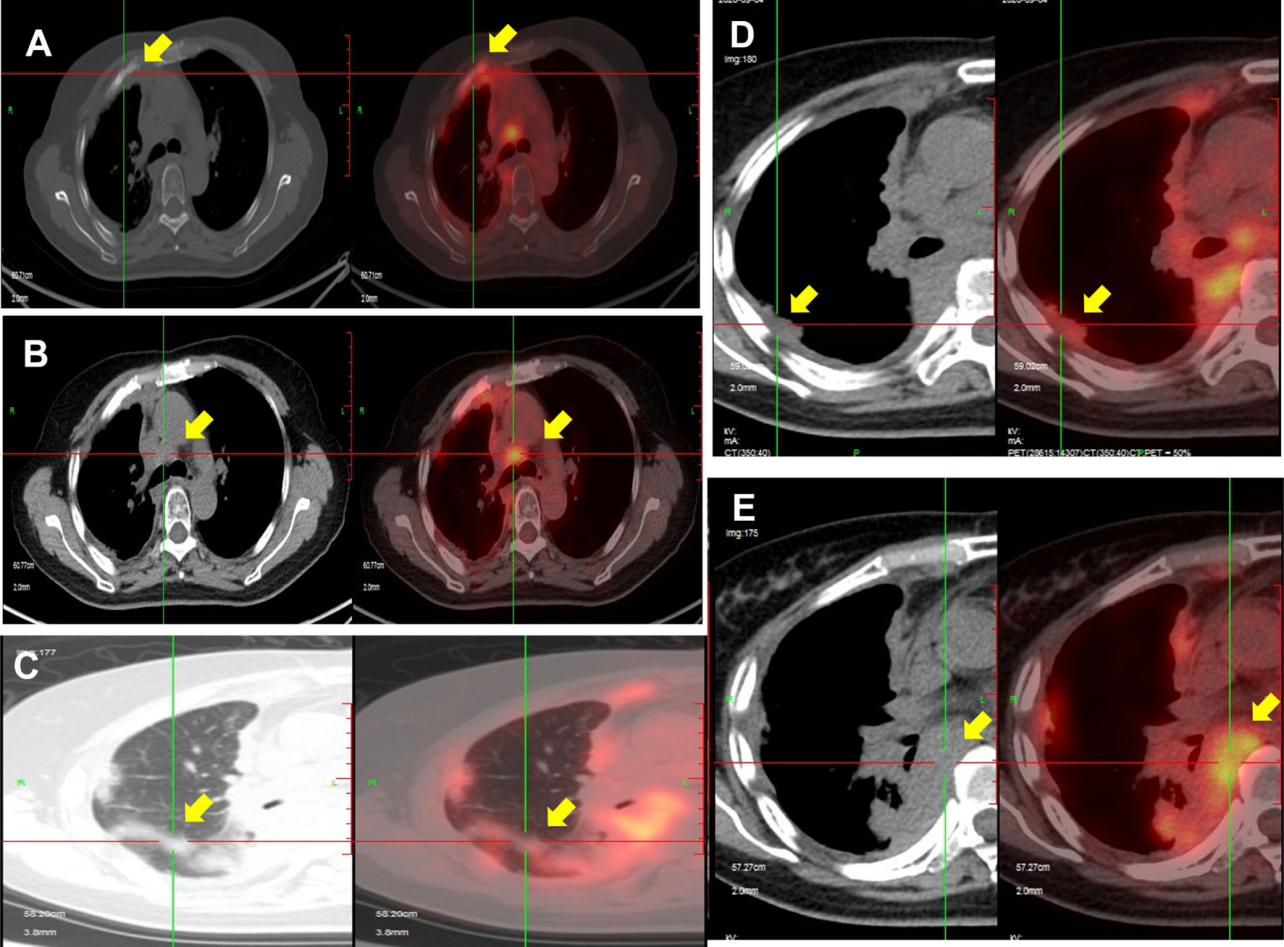
PET/CT scan in September 2020 showing disease progression after radiotherapy. Bone metastases **(A)**, lymph nodes in the mediastinum and diaphragm **(B, E)**, lung metastases **(C)**, and pleural metastases **(D)** show progression compared with scans in April 2020.

A PET/CT examination performed at our hospital in April 2020 showed that compared with January 2019, there were more nodules in the right lung and right interlobular fissure. The largest nodule was about 2.7 × 1.2 cm (previously 1.6 × 1.4 cm), and the F-fluorodeoxyglucose (FDG) uptake was increased with a maximum standardized uptake value (SUVmax) of 6.6 (previously 2.7). The anterior cortexes of the right second and third ribs were irregular, the lateral pleura was irregularly thickened with a larger range than before, and the FDG uptake was increased with an SUVmax of 7.6 (previously 3.3). A biopsy and lenvatinib treatment were recommended, but the patient refused after careful consideration. In September 2020, a PET/CT scan showed that the lung, pleura, and bone metastases were all progressing (**Figure 1**). Lenvatinib treatment was recommended again, but the patient still refused. In December 2020, the patient developed a worsening cough and started treatment with lenvatinib 4 mg once daily. This initial low dose of lenvatinib was recommended due to the patient’s low weight (<60 kg) and history of hypertension and arrhythmia.

**Figure 2:**
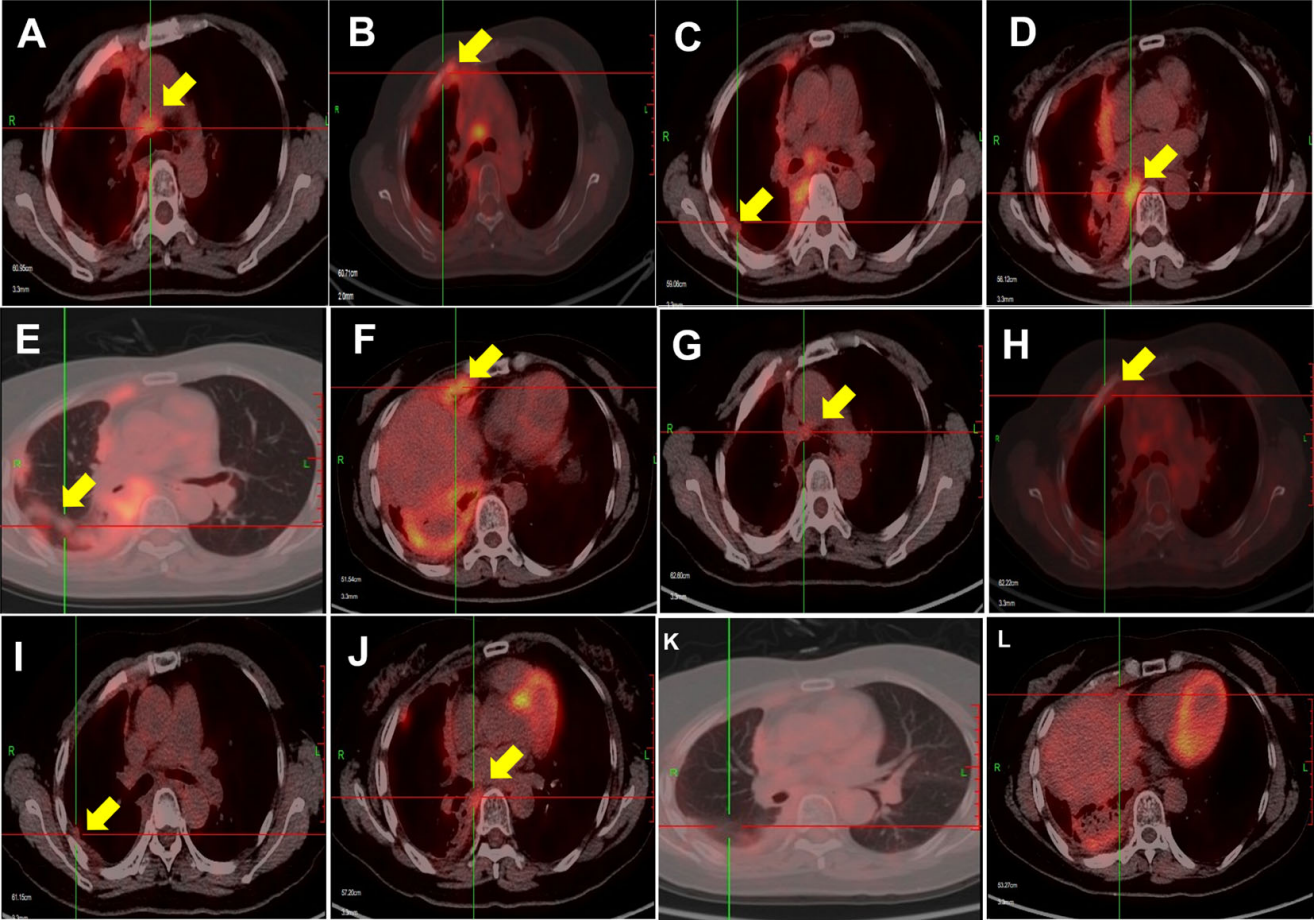
PET/CT scans showing disease response to lenvatinib treatment. The lesions in the right lung and right pleura were substantially smaller, and the metabolism of the lung, pleural, and bone metastases was decreased in March 2021 **(G–L)** compared with that in September 2020 **(A–F)**.

After 3 months, the symptoms of cough and dyspnea were significantly relieved, and a PET/CT scan (**Figures 2G–L**) showed that compared with the pre-lenvatinib assessment (**Figures 2A–F**), the right lung and right interlobular fissure nodules had nearly disappeared, and the FDG uptake was decreased, with an SUVmax of 2.7 (previously 5.5). FDG uptake in the ribs, right pleura, and mediastinal lymph nodes was also lower than that before lenvatinib treatment, with an SUVmax of 2.9 (previously 4.0), 6.2 (previously 8.3), and 3.7 (previously 6.4), respectively. The patient was evaluated as having a partial response to lenvatinib treatment.

**Figure 3:**
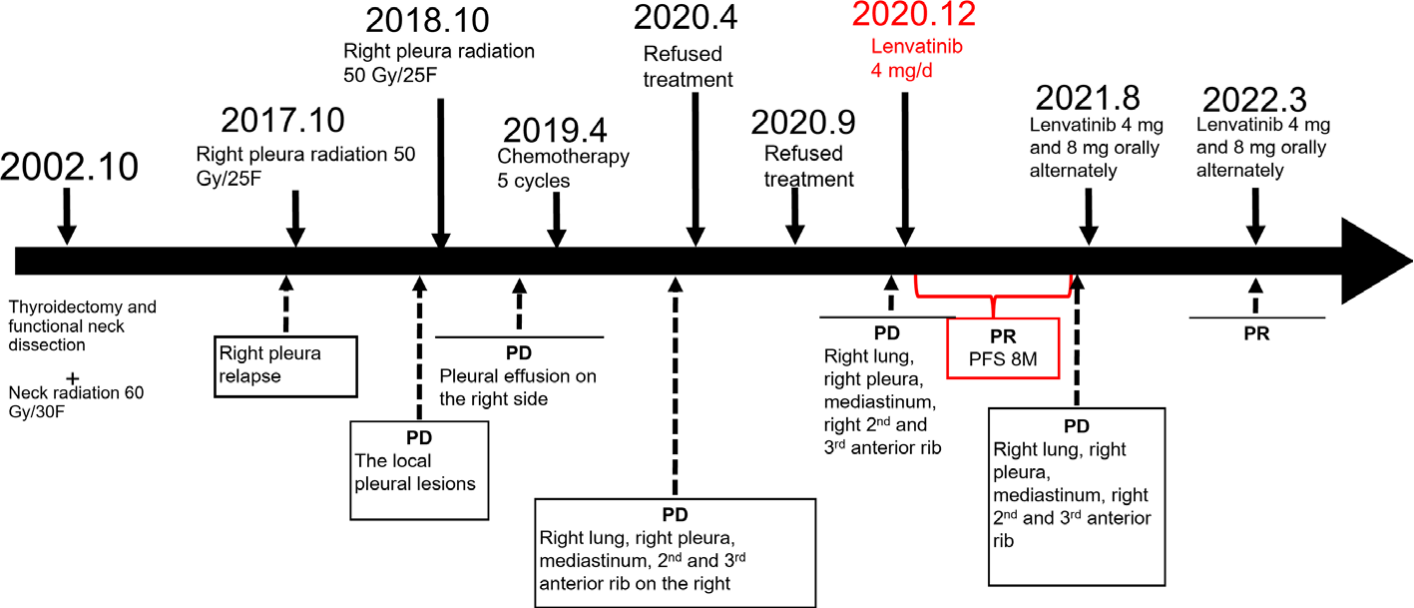
Summary of the treatment timeline of this case.

In the subsequent follow-up, the patient had a mild cough and no dyspnea and continued to respond to lenvatinib treatment. During lenvatinib treatment, the patient’s blood pressure was well controlled, her arrhythmia did not worsen, and there were no adverse reactions such as bleeding and diarrhea. In August 2021, the patient’s dyspnea worsened, and a PET/CT examination showed that, compared with March 2021, there were more nodules in the right lung and right interlobular fissure, and the FDG uptake was increased with an SUVmax of 6.4 (previously 2.7). The lateral pleura was irregularly thickened with a larger range than before, and the FDG uptake was increased with an SUVmax of 13.0 (previously 6.2). There were more lymph nodes in the mediastinum and the right side of the heart and diaphragm, and the FDG uptake was higher than before with an SUVmax of 6.6 (previously 3.7). Based on the above examination, we concluded that the disease had progressed, and the progression-free survival time was 8 months. The patient was advised to increase the lenvatinib dose. Currently, the patient takes lenvatinib once daily alternating between a 4- and 8-mg dose, and the symptoms are well controlled. The timeline for treatments and efficacy evaluations in this case is summarized in **Figure 3**.

**Figure 4:**
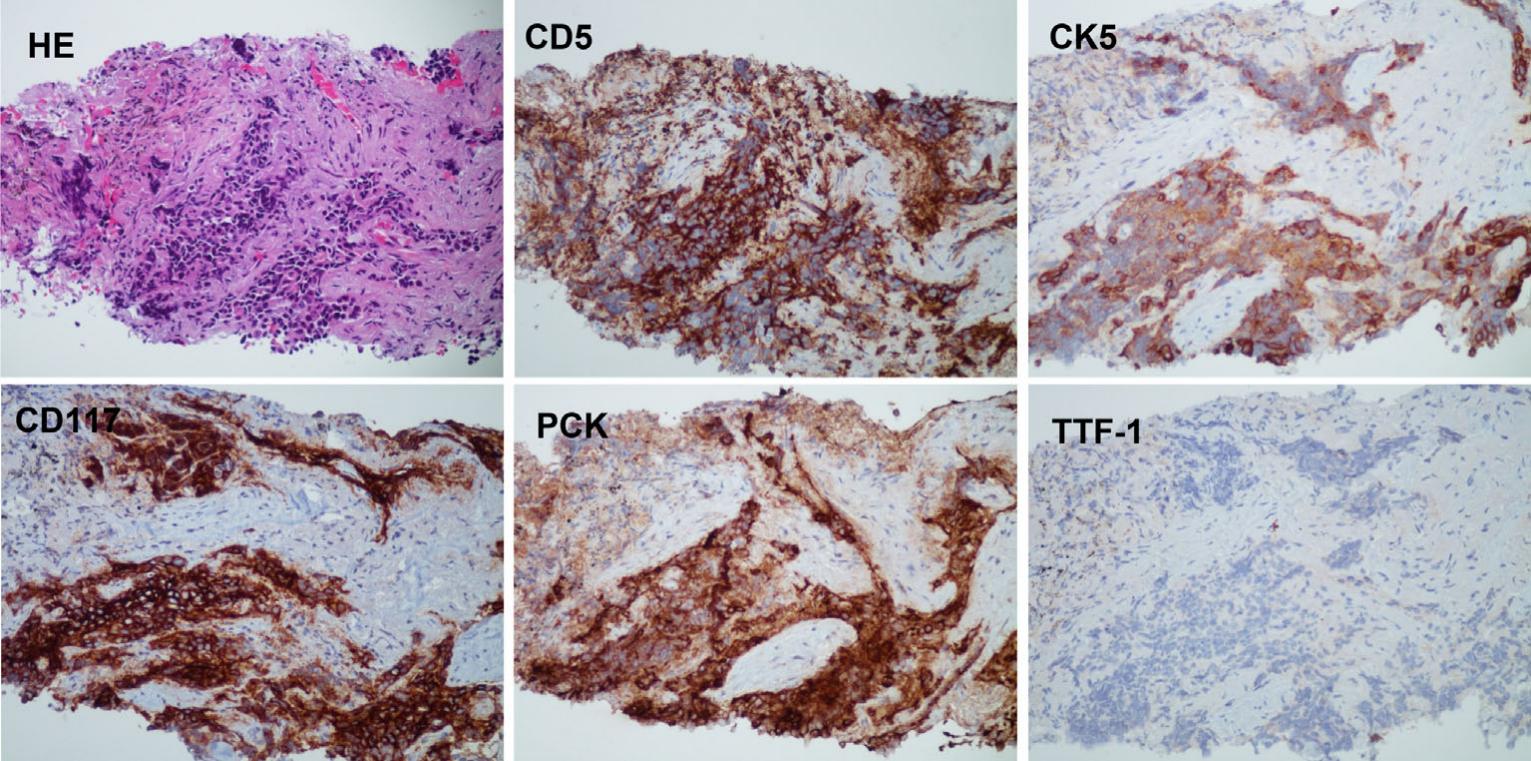
The histology and IHC staining of pleural nodule biopsy. The pathology showed that the immunohistochemical phenotype of tumor tissue supported the characteristics of thymic epithelial differentiation. Immunohistochemistry: CD5 (+), CK5 (+), CD117 (+), PCK (+), and TTF-1 (−).

Pleural nodule biopsy performed on 24 February 2022 showed that the immunohistochemical phenotype of the tumor tissue was consistent with the characteristics of thymic epithelial differentiation, which, combined with medical history, supported a diagnosis of secondary thymic adenocarcinoma. Immunohistochemistry staining was positive for CD5, CK5, CD117, and PCK and negative for TTF-1 (**Figure 4**).

## Literature review and discussion

Thymus tumors are rare, accounting for less than 1% of all cancers, with a largely unknown etiology and complex biology (11). ITTC is an independent clinicopathologic subcategory of thyroid tumor, which has a histological and immunophenotypic resemblance to thymic carcinoma (6). There are three recognized histological subtypes of ITTC, namely, squamous cell carcinoma type, lymphoepithelioma or basaloid type, and neuroendocrine carcinoma type (12). The patient in this case belonged to the squamous cell carcinoma type. The morphological diagnosis of ITTC, by fine-needle aspiration cytology or histopathology, is challenging because it is difficult to distinguish this disease from poorly differentiated carcinoma, squamous cell carcinoma, or anaplastic thyroid carcinoma. Studies have indicated that immunohistochemical staining with CD5, a marker of carcinoma of thymic origin, is useful in the histological diagnosis of this disease (13).

Given that the clinical features of ITTC are non-specific, a substantial proportion of patients already have invasion to adjacent soft tissue (60%) and metastases to regional lymph nodes (50%) at diagnosis (3, 6, 14, 15). Common metastatic sites include the brain, liver, and lungs (3). In the present case, our patient had pleural and lung metastases that caused dyspnea.

ITTC is relatively indolent and slow-growing compared with morphologically similar carcinomas, which often exhibit an aggressive clinical course and fatal outcome; thus, the clinical management differs substantially (2, 15). ITTC usually exhibits indolent biological behavior and is associated with a relatively favorable prognosis (15). Although the optimal treatment strategy remains uncertain, thyroidectomy and curative surgery, including resection of organs to which the tumor has extended, and systematic lymph node dissection should always be performed (16, 17). ITTC has been reported to be radiosensitive (18), and therefore, central compartment radiotherapy is a treatment option (6, 19). Surgery combined with adjuvant radiotherapy has been shown to improve the survival of patients with ITTC (7). Furthermore, Tsutsui et al. (6) reported that a patient who received radiotherapy after refusing surgery had complete tumor regression, without any local recurrence evident on CT assessments for at least 7 years. This clinical course demonstrates that ITTC is highly responsive to radiation, which enables long-term control with shrinkage of the tumor. Our patient initially underwent surgery with the aim of complete resection followed by radiotherapy and remained in remission for 15 years. However, after recurrence, the disease seemed resistant to radiotherapy. There have been few reports describing anticancer treatment for patients with recurrent or metastatic ITTC. Although chemotherapy is typically used in patients with locally advanced disease or gross residual disease post-surgery, studies have failed to show improved patient survival (6, 7). Considering the origin of ITTC, treatment of ITTC as a thymic carcinoma is a possible treatment strategy. Hence, in this case, we initially chose treatment with chemotherapy typically used for thymic carcinoma (carboplatin plus paclitaxel). However, after five cycles of chemotherapy, pleural effusion was controlled, but not the metastatic lesions.

Lenvatinib is a multitargeted TKI of VEGFR 1–3, FGFR 1–4, PDGFR-α, and the RET and c-KIT signaling networks, which are implicated in pathogenic angiogenesis, tumor growth, and cancer progression (20). In the phase III SELECT trial, lenvatinib prolonged progression-free survival and increased the overall response rate in patients with advanced RAI-refractory DTC (21). Consequently, the US Food and Drug Administration approved lenvatinib for the treatment of locally recurrent or metastatic, progressive, RAI-refractory DTC. The findings from REMORA, a single-arm, multicenter, phase 2 study of lenvatinib in patients with advanced or metastatic thymic carcinoma, have been reported at a median follow-up of 15.5 months (interquartile range 13.1–17.5) (22). Among 42 patients, 16 (38%) patients achieved a partial response, meeting the primary endpoint. These results support lenvatinib as a potential treatment option for patients with previously treated advanced or metastatic thymic carcinoma. However, there are no currently available data on the use of lenvatinib for the treatment of ITTC.

In the present case, lenvatinib treatment was initiated for an imminently life-threatening disease with right pleural effusion, diffuse lung metastases, and a rapidly progressing tumor burden. Disease control was not achieved with local radiotherapy, and the efficacy of systemic chemotherapy was limited. Lenvatinib treatment conferred a distinct benefit, effectively reducing the disease burden and slowing tumor growth.

There are several other multitarget tyrosine kinase inhibitors such as lucitanib, sorafenib, and cabozantinib that have shown efficacy in clinical trials that included patients with thymic tumors as well as thyroid tumors. For example, the results of the COSMIC-311 (23) trial revealed that cabozantinib significantly prolonged progression-free survival and may provide a new treatment option for patients with radioiodine-refractory DTC. Lucitanib, a potent oral inhibitor of fibroblast growth factor receptor types 1 and 2 (FGFR), vascular endothelial growth factor receptor types 1, 2, and 3 (VEGFR), and platelet-derived growth factor receptor types α and β (PGFRα/β), has also shown promising efficacy and a manageable side-effect profile in advanced solid tumors (including thymic and thyroid tumors) in a phase I/IIa study (24).

From the available information for the patient described in this case, we cannot evaluate whether the initial dose of lenvatinib was adequate. We selected lenvatinib 4 mg/day as the initial dose due to the low body weight of the patient and her complications and subsequently increased it to 8 mg/day. Although dosing should be modified according to the complications and general condition of a patient, the lower dose selected for this patient may not have been optimal. In contrast, the REMORA study of lenvatinib for thymic carcinoma in Japanese patients used an initial dose of lenvatinib of 24 mg/day. In addition, the randomized COSMIC-311 trial compared lenvatinib 24 and 18 mg for patients with radioactive iodine-refractory differentiated thyroid cancer and showed inferior outcomes with the lower 18-mg dose (23). In the COSMIC-311 trial, it was possible to manage hypertension occurring during lenvatinib treatment through adequate treatment interruptions and dose reductions from the initial 24 mg/day dose as necessary. Therefore, it may be optimal to start treatment with a higher initial dose with sufficient supportive care for as long as possible.

## Conclusion

The management of ITTC is challenging due to the rarity of this malignancy and the limited evidence available. In this case study, a patient with ITTC was successfully treated with lenvatinib salvage therapy. Although the patient had widespread metastases and a very poor prognosis, lenvatinib treatment resulted in a durable reduction in tumor burden and maintenance of quality of life.

## Data availability statement

The original contributions presented in the study are included in the article/supplementary material. Further inquiries can be directed to the corresponding author.

## Ethics statement

The studies involving human participants were reviewed and approved by the Institutional Review Board of Tongji Hospital. The patients/participants provided their written informed consent to participate in this study.

## Author contributions

YY and QL composed the manuscript and literature review. CK and GZ provided the figures and conducted the pathology review. YY, QL, and JZ were responsible for the acquisition, analysis, or interpretation of data for the work, revising it critically for important intellectual content, and gave final approval of the version to be published. All authors contributed to the article and approved the submitted version.

## Acknowledgments

We thank the members of the research group for their discussion and guidance on the diagnosis and treatment of the patient.

## Conflict of interest

The authors declare that the research was conducted in the absence of any commercial or financial relationships that could be construed as a potential conflict of interest.

## Publisher’s note

All claims expressed in this article are solely those of the authors and do not necessarily represent those of their affiliated organizations, or those of the publisher, the editors and the reviewers. Any product that may be evaluated in this article, or claim that may be made by its manufacturer, is not guaranteed or endorsed by the publisher.
